# Antiseptic Vaginal and Intra-Uterine Injections Unnecessary, If Not Injurious in the Daily Practice of Obstetrics

**Published:** 1893-10

**Authors:** Darwin Colvin

**Affiliations:** Clyde, N. Y.


### ANTISEPTIC VAGINAL AND INTRA-UTERINE INJECTIONS UNNECESSARY, IF NOT INJURIOUS IN THE DAILY PRACTICE OF OBSTETRICS.

BY DARWIN COLVIN, M. D., CLYDE, N. Y.

I am well aware that it requires some courage to stand before this intelligent body of medical men, as an opponent of the practice of using antiseptic vaginal and intra-uterine injections in the every-day practice of midwifery. However, the extreme views which were so liberally paraded in the journals, but a short time since, are now seldom reiterated for want of conversions.

The practice of frequent flushings of the vagina with antiseptic solutions when only normal processes are going on, during, and immediately after parturition, has begun its march, and is rapidly moving on to join that innumerable caravan of other ephemeral fashions which have preceded it. What good reason can the accoucheur give for introducing poisonous solutions upon mucous tissues, which are in a healthy condition?

Such conduct reminds me of a story of my boyhood: A stern parent was asked how he governed his boy. He replied, "I always punish him whenever I see him, for I know he has just come out of, or is just going into, mischief." To make the application :—the accoucheur will punish with or without proper justification. I shall have nothing to say about antiseptic precautions in the various surgical procedures of the present day; it is only against the unnecessary, and oftentimes dangerous interference with normal physiological processes, that I desire to enter a protest.

I will not consume your time in quoting authorities for or against my position, as they are all familiar to you, but I shall base my conclusions upon the results of more than two scores of years of constant obstetric practice, together with the care of between sixty and seventy cases since that branch of general practice has been largely abandoned. It has appeared to me when reading the various contributions of those who write so learnedly on the necessity of extreme antiseptic measures in parturition, that they fail to make use of a reasonable amount of what all practitioners should possess—good common sense.

Do they ask themselves these very important questions: Is the mortality of lying-in women any less by such means? Is the death rate any less than it was fifty years ago? Assuredly they cannot reply in the affirmative.

All advocates of that complete metamorphosis of the surroundings of the accoucheur and his patient, which are so ludicrously emphasized in their writings, cannot but be ignorant of the results obtained in the practice of midwifery half century ago, when the percentage of recoveries was just as great as at the present day. True, there was a less number of cases of cervical fissures and perinaeal lacerations fifty years ago than at the present time; but that can be readily explained by the more frequent and unskillful use of the forceps.

If then, the mortality is no less where there is so much useless interference, why, I ask, is so much required? All should require careful, thorough, and complete cleanliness, but do we always have that important factor to aid us in the patient's recovery? When I think of the scenes and surroundings of my early obstetric experience, I am almost led to say that even cleanliness is scarcely necessary to secure to the parturient female a quick recovery. I have spent hour after hour in shanties and log houses, in which I would not now consider it safe to keep my horses ; places where, in the winter, the snow could be seen sifting through the crevices, and where, notwithstanding the log fire, my fingers would be so benumbed with cold, that I could neither make a digital examination, nor ligate the funis, until my fingers had been first immersed in hot water.

No persons were at hand, to care for the patient, save some temporarily invited neighbors, and no injections were used, yet, the patient recovered. There could have been no nomadic tribes of germs in that day, for in connection with this unpropitious state of things, I have often found upon my next visit, an almost unbearable odor arising from the bed-clothing. Frequently, upon making further investigations, I would find that the external genitals had had no introduction to soap and water, and that the exsiccated blood had been allowed to remain upon the parts, for fear, if removed, that "she would catch cold."

Added to this evidence of ignorance, was the belief, quite frequently expressed, and as often carried out, that the placenta should be burned "to prevent a broken breast." Fortunately, the immense fireplace afforded every facility for its cremation.

While writing this paper, my memory goes back to a score or more of abortions, many of them occurring in the unmarried, where for the purpose of concealment, no physician was at first called in, and where not even ordinary cleanliness was used, for fear of exposure; yet in none of them was an antiseptic injection used, and all, in which the placenta had been expelled, recovered. For many years I was familiar with this state of things—and such a thing as a fatal termination after parturition at term, scarcely entered my mind.

True, at long intervals, I would hear of a death from puerperal, or what was then called child-bed fever; yet I had been in practice more than ten years before I saw a case, and that was in consultation. Since that time I have only seen three, these also being seen in consultation. Two of these had been treated by the fashionable antiseptic injections, but how thoroughly I do not now remember; yet all died, though they were treated by very clever practitioners.

I have performed seventeen craniotomies in my own, and the practice of others, and although no antiseptic injections were used, and frequently even cleanliness was not observed, all of the women recovered.

Throughout the early period of my professional work, there was scarcely one who pretended to be a nurse, and if perchance one was found, she was generally some impecunious relative or neighboring girl, who was expected to do the work of the house, care for the other children, and possibly milk a cow or two, besides attending to the patient and baby for the stereotyped *nine days*. At the expiration of this time, the so-called nurse was disposed of, and the mother again took the helm, and went on rapidly to recovery.

Now, some may say that "necessity drove this woman from her bed," but this I deny, for necessity does not guarantee the patient against disease.

Picture to yourselves a highly sensitive, intelligent young *primipara* watching the elaborate arrangements being made for conducting her labor according to the most approved antiseptic methods. Would it be strange for her to exclaim: "Doctor, has that God whom I have been taught is a synonym of love, after requiring of me obedience to his command to multiply and replenish the earth, placed my life in such jeopardy, as to require you at this time to thus fortify yourself as an antagonist of death? Why am I in greater danger than was the mother who bore me?"

Every physician should be governed in his treatment of cases by the results. If, in the management of a large number, the results have been perfectly satisfactory without antiseptic injections, how can he expect that a meddlesome change will be of service? Strange as it may seem to some, my records show that without the use of any antiseptic injections, I have delivered at term, more than twelve hundred babies, with but one maternal death, and that one occurred after an easy labor, and within fifteen minutes after the extrusion of the placenta.

I have never been able to explain her death. Now, this extremely favorable showing was not due to the fact that these patients were living in the country, as one of our Fellows remarked when criticising my former paper: Why, may I ask, should we resort to agents, which, if they could do no harm—and who can say they will not—certainly cannot aid us in improving upon the past?

In an article written by a distinguished gynaecologist of this country, on the subject of antiseptic injections during and after labor, he says: "In private practice, in the homes of comfort, with the attendance of the best practitioners, and the care of good nurses, many a young mother yields her life to the dangers which still accompany home confinement. We are told that the records of a prominent insurance company, reveal a mortality of 17 *per cent. in private practice, and that among the better class*" (italics mine). No greater slander, and I might almost say, untruth, was ever written. I believe statistics will justify me in saying, that the mortality in country practice, and without antiseptics, is *not one per cent.*

Another more radical advocate of antiseptics in midwifery, who is quite inclined to question the intelligence, and even the veracity of the country practitioner, says: "I believe that by far the greatest number of general practitioners yet conduct their deliveries in the same way that they learned at college many years ago, and which they have been accustomed to follow since they began to practice." After having criticised the intelligence of those physicians who are not converts to strict antisepsis in midwifery, and who claim a small percentage of deaths in their practice, he says: "Either their memory fails them, or they have a convenient definition of puerperal fever, in consequence of which puerperae die of peritonitis, metritis, pneumonia, pleurisy, heart disease, liver complaint, kidney trouble, meningitis, typhoid fever, etc., but never of a disease, the mere mention of which, septicaemia, would touch the propriety of adopting the antiseptic treatment."

Poor country doctors, and those of cities who do not choose to follow any man's dictation in the use of antiseptic agents in obstetric practice, when will you cease to be so ignorant as not to be able to diagnose puerperal fever from pneumonia, or heart disease? How forcibly does this writer exemplify the familiar proverb—"A little learning is a dangerous thing!"

To those who have been taught that the genital organs of every parturient woman are surrounded by germs, which are ever ready to pounce upon them when they begin that normal physiological work which the Almighty requires for the purpose of peopling the earth, let me say: Do not be startled, but abandon your prejudices, and the future will hear you exclaim—"the otherwise competent physician requires only complete asepsis of the female genital organs after delivery, to make him a successful obstretrician."—*Trans. N. Y., State Med. Asso.* 1893.

---

A NEW MEDICAL DICTIONARY.—A completely new Medical Dictionary is announced for early publication by Lea Brothers & Co. The author, Dr. Alexander Duane, of New York, is already widely known as the medical expert for Webster's International Dictionary. His new work has been drafted to supply medical students with all desired information concerning the words they will meet in their course of reading, and as the vocabulary has been selected most liberally, the work will be of value to practitioners also. The pronunciation of each word is given by a simple and obvious phonetic spelling; then follows the derivation, an unexcelled aid to memory, and finally a full definition. Descriptive matter has been appended to such words as cannot be adequately explained by simple definition. Thus diseases are described, and their symptoms and treatment are given; drugs are followed by their properties, effects, doses, etc. Extensive tables of bacteria, doses, etc., are placed in the alphabet most conveniently for reference. A work of real value is promised, and we shall take an early opportunity of reviewing it in this journal.

---

The danger to life from burns, Prof. Chapman says, depends more on their extent than upon their depth.—*Ex.*

